# Alteration of the Sitting and Standing Movement in Adult Spinal Deformity

**DOI:** 10.3389/fbioe.2021.751193

**Published:** 2022-01-13

**Authors:** Eddy Saad, Karl Semaan, Georges Kawkabani, Abir Massaad, Renee Maria Salibv, Mario Mekhael, Marc Fakhoury, Krystel Abi Karam, Elena Jaber, Ismat Ghanem, Virginie Lafage, Wafa Skalli, Rami Rachkidi, Ayman Assi

**Affiliations:** ^1^ Faculty of Medicine, University of Saint-Joseph, Beirut, Lebanon; ^2^ Orthopaedics Surgery, Lenox Hill Hospital, New York, NY, United States; ^3^ Institut de Biomécanique Humaine Georges Charpak, Arts et Métiers, Paris, France

**Keywords:** adult spinal deformity (ASD), kinematics, sitting, standing, radiograph assessment, movement analysis, quality of life

## Abstract

Adults with spinal deformity (ASD) are known to have spinal malalignment affecting their quality of life and daily life activities. While walking kinematics were shown to be altered in ASD, other functional activities are yet to be evaluated such as sitting and standing, which are essential for patients’ autonomy and quality of life perception. In this cross-sectional study, 93 ASD subjects (50 ± 20 years; 71 F) age and sex matched to 31 controls (45 ± 15 years; 18 F) underwent biplanar radiographic imaging with subsequent calculation of standing radiographic spinopelvic parameters. All subjects filled HRQOL questionnaires such as SF36 and ODI. ASD were further divided into 34 ASD-sag (with PT > 25° and/or SVA >5 cm and/or PI-LL >10°), 32 ASD-hyperTK (with only TK >60°), and 27 ASD-front (with only frontal malalignment: Cobb >20°). All subjects underwent 3D motion analysis during the sit-to-stand and stand-to-sit movements. The range of motion (ROM) and mean values of pelvis, lower limbs, thorax, head, and spinal segments were calculated on the kinematic waveforms. Kinematics were compared between groups and correlations to radiographic and HRQOL scores were computed. During sit-to-stand and stand-to-sit movements, ASD-sag had decreased pelvic anteversion (12.2 vs 15.2°), hip flexion (53.0 vs 62.2°), sagittal mobility in knees (87.1 vs 93.9°), and lumbar mobility (L1L3-L3L5: −9.1 vs −6.8°, all *p* < 0.05) compared with controls. ASD-hyperTK showed increased dynamic lordosis (L1L3–L3L5: −9.1 vs −6.8°), segmental thoracic kyphosis (T2T10–T10L1: 32.0 vs 17.2°, C7T2–T2T10: 30.4 vs 17.7°), and thoracolumbar extension (T10L1–L1L3: −12.4 vs −5.5°, all *p* < 0.05) compared with controls. They also had increased mobility at the thoracolumbar and upper-thoracic spine. Both ASD-sag and ASD-hyperTK maintained a flexed trunk, an extended head along with an increased trunk and head sagittal ROM. Kinematic alterations were correlated to radiographic parameters and HRQOL scores. Even after controlling for demographic factors, dynamic trunk flexion was determined by TK and PI-LL mismatch (adj. *R* ^2^ = 0.44). Lumbar sagittal ROM was determined by PI-LL mismatch (adj. *R* ^2^ = 0.13). In conclusion, the type of spinal deformity in ASD seems to determine the strategy used for sitting and standing. Future studies should evaluate whether surgical correction of the deformity could restore sitting and standing kinematics and ultimately improve quality of life.

## 1 Introduction

With the aging of the population, degenerative diseases have been increasing in prevalence (Safaee et al., 2020) and placing a significant burden on the healthcare system (Pellisé et al., 2015).

Adult spinal deformity (ASD) encompasses a multitude of pathological entities mainly resulting from a primary degenerative process, but also from trauma or progression of a pathology of the spine such as adolescent idiopathic scoliosis or Scheuermann’s hyperkyphosis (Aebi, 2005). These seemingly heterogeneous diseases all demonstrate postural malalignment defined by the presence of at least one of the following radiographic criteria: pelvic tilt (PT) > 25°, sagittal vertical axis (SVA) > 50 mm, coronal Cobb angle > 20°, thoracic kyphosis (TK) > 60°, and pelvic incidence–lumbar lordosis (PI-LL) mismatch > 10°, according to the International Spine Study Group (Schwab et al., 2012; Bakhsheshian et al., 2017; Kim et al., 2017).

Adults with spinal deformity are known to present with spinal malalignment and recruit compensation mechanisms at the hips and knees to maintain balance (Le Huec et al., 2019). These radiographic alterations and compensation strategies have been shown to affect the patients’ health-related quality of life (HRQOL) and limit their daily life activities (Christopher Kieser and Wyatt, 2019). In fact, it is estimated that ASD have some of the most impaired HRQOL scores among all chronic diseases (Pellisé et al., 2015).

Motion analysis is increasingly being used to assess functionality in ASD subjects. Kawkabani et al. have shown that ASD subjects walked slower with an increased double support phase, and maintained a flexed attitude in their thorax, hips, and knees while walking (Kawkabani et al., 2021). Severijns et al. also described a similar finding in ASD subjects with a decompensated sagittal deformity (Severijns et al., 2021). Although walking is an essential activity in daily life, limitations in other activities are usually observed when collecting HRQOL outcomes. Thus, kinematics of daily life activities, other than walking, should be assessed in ASD to better address the quality-of-life concerns in these patients.

Sitting and standing represent important life activities that are commonly used during the day. To be fully functional, an individual must hold the sitting position for a long time while being able to transition from the sitting to the standing position and vice versa. In fact, an alteration of this activity in the elderly was a predictor of dependence, institutionalization, and even mortality (Hirvensalo et al., 2000; Yamada and Demura, 2009). Furthermore, sitting and standing constitute complex tasks requiring fine musculoskeletal coordination and thus are expected to be affected in patients with spinal deformity (Sadeghi et al., 2013).

Few studies have previously explored alterations of the sit-to-stand movement in ASD. In particular, Bailey et al. used a 3D depth-camera to describe motion kinematics and kinetics from 15 ASD patients, both pre- and postoperatively, compared with 10 controls. They showed that ASD patients had increased peak sagittal vertical axis (SVA) during sit-to-stand as well as increased lumbar and lower limb torques, which could be corrected by surgical interventions (Bailey et al., 2019). However, the segmental motion of the spine was not analyzed and further subdivision of the ASD population according to the type of spinal deformity was not possible due to the small sample size.

Therefore, the aim of our study was to evaluate pelvis, lower limb, trunk, spinal segment, and head kinematics in ASD subjects with different types of spinal deformity, during the sit-to-stand and stand-to-sit movements as well as their relationships with their HRQOL scores and radiographic parameters.

## 2 Materials and Methods

### 2.1 Study Design

This is a cross-sectional IRB-approved study (CEHDF1259) evaluating kinematic alterations in different subgroups of ASD subjects compared with controls. All participants signed an informed consent prior to the trials.

### 2.2 Participants

ASD subjects were referred to our laboratory by their physicians for pain and/or disability. Inclusion criteria included the presence of at least one of the following radiographic alterations: pelvic tilt (PT) >25°, sagittal vertical axis (SVA) >50 mm, pelvic incidence–lumbar lordosis mismatch (PI-LL) >10°, thoracic kyphosis (TK) >60°, and/or coronal Cobb angle >20° (Schwab et al., 2012; Kim et al., 2017), as well as being older than 20 years and reporting pain or discomfort. Subjects presenting with other motion altering disorders (neurological, rheumatic, infectious, tumoral, or other diseases) or a history of spine or lower limb surgery were excluded from our study.

Asymptomatic control subjects were recruited following a call that was made within our institution. Inclusion criteria included being at least 20 years old. Exclusion criteria included the presence of longstanding pain or disability, musculoskeletal (adolescent idiopathic scoliosis, Scheuermann’s disease … ) or neurological (spinal stenosis, sciatica … ) disorders, acute injury, prior history of spine or lower limb surgery, and the presence of one of the radiographic criteria that characterize ASD.

Mild degenerative modifications were not considered as exclusion criteria if they did not cause clinical manifestations (pain and/or disability) since some degree of degenerative changes is inevitable with age.

### 2.3 Data Acquisition

#### 2.3.1 Demographics

Age (year), sex (F/M), height (cm), weight (kg), and BMI (kg/m^2^) were collected for each subject.

#### 2.3.2 Radiographic Parameters

**FIGURE 1:**
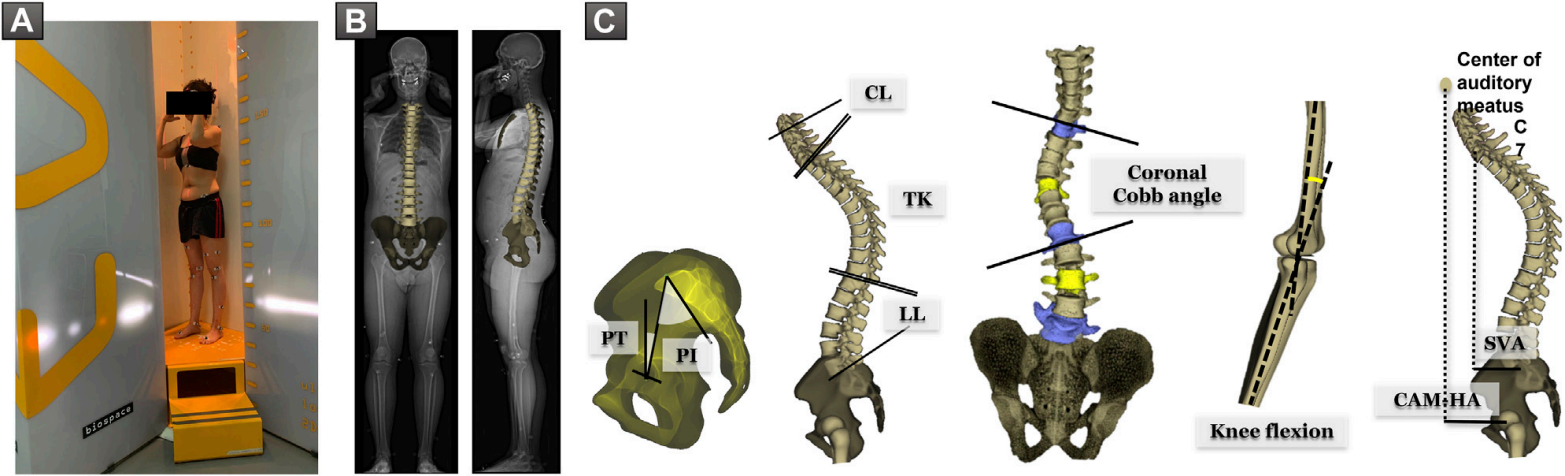
**(A)** Subject in the free-standing position during acquisition of EOS biplanar X-rays. **(B)** 3D reconstruction of the spine and pelvis. **(C)** Spino-pelvic and postural parameters: pelvic incidence PI (°), pelvic tilt PT (°), L1S1 lumbar lordosis LL (°), T1T12 thoracic kyphosis TK (°), C2C7 cervical lordosis CL (°), knee flexion (°), coronal Cobb angle (°), sagittal vertical axis SVA (mm), and distance from center of auditory meatus plumb line to hip-axis CAM-HA (mm).

All subjects underwent low dose EOS full-body biplanar X-rays in the free-standing position (Chaibi et al., 2012) (EOS Imaging, Paris, France) (Figure 1A). Three-dimensional reconstructions of the subjects’ spine, pelvis, and lower limbs were performed using Stereos (v1.8.99.20R) (Figure 1B). The following spino-pelvic were extracted from the 3D reconstructions: pelvic incidence PI (°), radiographic pelvic tilt PT (°), L1S1 lumbar lordosis LL (°), PI–LL mismatch (°), T1T12 thoracic kyphosis TK (°), coronal Cobb angle (°), and knee flexion (°). C2C7 cervical lordosis CL (°), sagittal vertical axis SVA (mm), and distance from center of auditory meatus plumb line to hip-axis CAM-HA (mm) were extracted from standing radiographs (Figure 1C).

Based on their radiographic alterations, ASD subjects were subdivided into three groups:

ASD-sag: presenting with a sagittal malalignment: SVA > 50 mm and/or PT > 25° and/or PI-LL > 10°, irrespective of the presence of a coronal Cobb angle deformity or a thoracic hyperkyphosis;
ASD-hyperTK: presenting with only a thoracic hyperkyphosis TK > 60°;
ASD-front: presenting with only a Coronal Cobb angle > 20°.

#### 2.3.3 HRQOL Questionnaires

All subjects filled the following HRQOL questionnaires:

SF-36 with both its physical (PCS) and mental (MCS) components, on a scale of 0–100, decreasing with severity, and normalized to the local population;
Oswestry Disability Index (ODI) measures disability on a scale of 0–100, increasing with severity;
Beck’s Depression Inventory (BDI) evaluates depression, on a scale of 0–63, increasing with severity;
Visual Analog Scale (VAS) measures pain intensity on a scale of 0–10, increasing with severity.

#### 2.3.4 Motion Analysis

**FIGURE 2:**
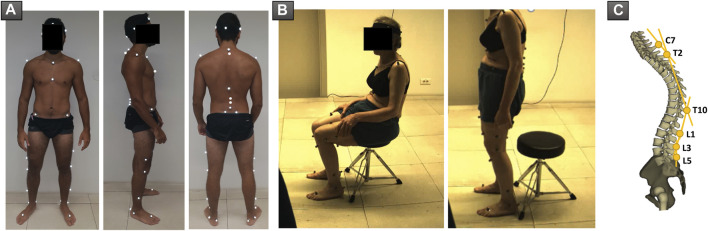
**(A)** Positioning of markers used during acquisition of sit-to-stand and stand-to-sit movements. **(B)** Patient during acquisition, in the sitting and standing positions, respectively. **(C)** Representation of spine segments as described by Leardini et al.

Motion capture was performed using the Vicon opto-electronic system (Vicon Motion Systems, Oxford, UK). The acquisition was completed using eight infrared cameras (Vero 2.2, 200 Hz) and two front and side video cameras. A calibration was carried out before each acquisition. Forty-one markers were used, four of which were placed on a band positioned on the patient’s head. Lower limb markers were placed according to the Davis protocol (Davis et al., 1991) on the following structures: anterosuperior and posterosuperior iliac spines, distal third of the femur, lateral knee condyles, distal third of the tibia, lateral malleoli, calcaneum, and base of second metatarsal. Trunk and spine markers were positioned according to the Leardini protocol (Leardini et al., 2011) on the following bony landmarks: both acromions, suprasternal notch, xiphoid process, and spinous processes of C7, T2, T10, L1, L3, and L5 vertebrae (Figure 2A).

Subjects were asked to sit on a backless stool, with their feet flat on the ground. The height of the seat was adjusted so that hips and knees were both at 90°. Subjects were then instructed to stand up without support, while looking straight ahead. In case they were not able to stand on their own, they were allowed to lean on their thighs. Subjects had to remain upright for 5 s before sitting down again in their seat, while keeping the gaze straight ahead and without leaning. Subjects were excluded when markers, especially those of the pelvis, were not visible during motion tracking (Figure 2B).

The position of each marker was verified on the standing biplanar radiographs and, if necessary, the 3D coordinates of the marker in its correct anatomical position were measured on standing radiographs in the corresponding body segment’s frame of reference and used to reconstruct the marker on ProCalc (Vicon, Oxford, UK) to correspond to the anatomical landmark. The position of the markers was instantly detected in the room’s frame of reference and enabled the reconstruction of the various body segments: the head, trunk, pelvis, and the segments of the lower limbs, using Nexus and ProCalc (Vicon). The motion of each segment relative to the other allowed the extraction of kinematic curves for each joint in the three planes of space. The motions of the head, trunk, and pelvis were also calculated in the room’s frame of reference. This correction was applied only on markers within a rigid body segment (i.e., pelvis, head …) and not on isolated markers such as those of the spine since it is not possible to predict their accurate position relative to other markers during motion.

Segmental analysis of the spine was also performed using the following segments: L3L5, L1L3, T10L1, T2T10, and C7T2 (Figure 2C). The angles between adjacent segments were extracted in the sagittal plane. For example, for the angle between L1L3 and L3L5 representing lumbar lordosis, increasing values denote a loss of lumbar lordosis, while decreasing values signal an increased lumbar lordosis. In addition, the angle between the pelvis and the L3L5 segment was calculated.

A cycle for either the sit-to-stand or stand-to-sit movement was delimited starting the first frame before the beginning of the movement defined as the initial horizontal displacement of a head or trunk marker, until the next frame after the stop of the movement defined as the final frame where no further movement was detected along the trajectories of all markers. Oscillations, occurring before and after the sit-to-stand and stand-to-sit movements and defined by a reversal in motion direction of the markers, were excluded from the cycle. Cycles were then normalized between 0 and 100%.

Several trials were recorded for each subject. Consistency between trials was verified on the kinematic waveforms in Polygon (Vicon). One repeatable trial was selected for each subject and exported in Excel.

The kinematic curves of the various parameters allowed the extraction of the maximum, minimum, and mean values as well as the range of motion (ROM) corresponding to the difference between the two extremes, both during the sitting–standing and the standing–sitting transitions. Kinematic parameters were computed in Matlab (Mathworks, Natick, USA; R2016a).

### 2.4 Statistical Analysis

The distribution of all variables was assessed for normality using Shapiro–Wilk test. Since most parameters did not follow a normal distribution, nonparametric tests were used for statistical analysis.

Demographic parameters were compared between ASD and controls using Mann–Whitney test. Sex was compared using χ^2^ test.

HRQOL scores, and standing radiographic and kinematic parameters (mean, maximum, minimum, and range of motion ROM) during sit-to-stand and stand-to-sit movements were compared between ASD groups and controls using Kruskal–Wallis test followed by Conover–Iman pairwise comparisons.

The relationships between kinematic alterations and both radiographic parameters and HRQOL scores were investigated using Pearson’s correlation.

Determinants of kinematic alterations were explored using a multivariate analysis (stepwise multiple linear regression) with demographic and standing radiographic parameters as independent variables. Adjusted *R* ^2^, standardized β coefficients, and *p*-values were reported for each model.

Statistical analyses were performed using XLSTAT (version 2019; Addinsoft, Paris, France). The level of significance was set at 0.05 and adjusted by a Bonferroni correction when necessary.

## 3 Results

### 3.1 Demographics

In total, 93 ASD [50 ± 20 years old (20–85); 71 F and 22 M] and 31 controls [45 ± 15 years old (21–76); 18 F and 13 M] were included in our study. There were no significant differences in age or sex distribution between the two groups (*p* = 0.10 and *p* = 0.06 resp.). There was no statistically significant difference in weight nor BMI between both groups (weight: ASD: 71 ± 15 kg vs controls: 69 ± 13 kg, *p* = 0.63; BMI: ASD: 27 ± 5 kg/m^2^ vs controls: 25 ± 3 kg/m^2^, *p* = 0.21). ASD were on average 5 cm shorter than controls (ASD: 162 ± 10 cm vs controls: 167 ± 8 cm, *p* = 0.01).

ASD subjects were further divided into 34 ASD-sag, 32 ASD-hyperTK, and 27 ASD-front.

### 3.2 Standing Radiographic Parameters

**FIGURE 3:**
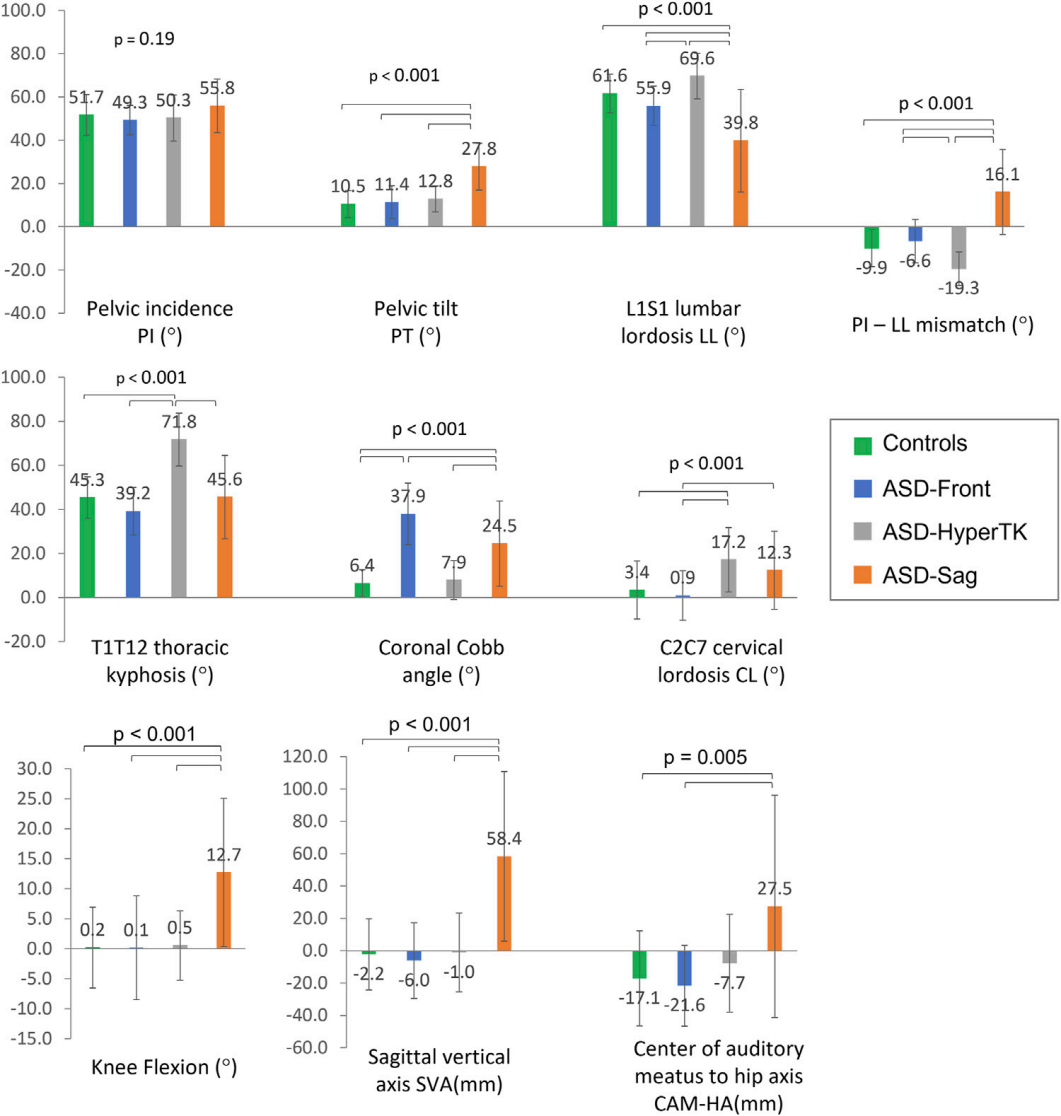
Comparison of spino-pelvic and postural parameters between subgroups: controls, ASD-front, ASD-hyperTK, and ASD-sag.

All groups had similar PI values (ASD: 52 ± 11° vs controls: 52 ± 10°, *p* = 0.19). As expected, ASD-sag had an increased SVA (58.4 ± 52.5 mm vs controls: −2.2 ± 22.1 mm, *p* < 0.001), CAM-HA (27.5 ± 68.7 mm vs controls: −17.1 ± 29.4 mm, *p* = 0.005), and PT (27.8 ± 10.8° vs controls: 10.5 ± 6.2°, *p* < 0.001) when compared with the other groups. They also presented with a decreased LL and an increased PI-LL mismatch (39.8 ± 23.7° and 16.1 ± 19.6° resp., *vs*. controls: 61.6 ± 9.0° and −9.9 ± 8.6° resp., both *p* < 0.001), as well as an increased knee flexion (12.7 ± 12.4° vs controls: 0.2 ± 6.8°, *p* < 0.001) when compared with other groups. ASD-hyperTK showed an increased TK (71.8 ± 12° vs controls: 45.3 ± 9.4°, *p* < 0.001), LL (69.6 ± 10.5° vs controls: 39.8 ± 23.7°, *p* < 0.001), and CL (17.2 ± 14.6° vs controls: 3.4 ± 13.2°, *p* < 0.001) compared with other groups. ASD-front presented with an increased coronal Cobb angle compared with other groups (37.9 ± 14.0° vs controls: 6.4 ± 6.2°, *p* < 0.001) (Figure 3).

### 3.3 HRQOL Scores

All ASD subjects showed altered HRQOL scores, with ASD-sag being the most affected, followed by ASD-hyperTK and then ASD-front. ASD-sag had a significantly decreased PCS (ASD-sag: 36.2 ± 8.1 vs controls: 50.1 ± 7.7, *p* < 0.001) and MCS (ASD-sag: 48.8 ± 10.1 vs controls: 55.0 ± 6.3, *p* = 0.005). They showed moderate levels of pain (VAS: 6.7 ± 2.6 vs 1.3 ± 0.7, *p* < 0.001), significantly increased disability (ODI: 38.0 ± 16.9 vs 3.2 ± 5.0, *p* < 0.001), and higher depression levels (BDI: 11.3 ± 10.0 vs 2.2 ± 3.8, *p* < 0.001) (Table 1).

**TABLE 1:** Comparison of health-related quality of life (HRQOL) scores between subgroups: controls, ASD-front, ASD-hyperTK, and ASD-sag.

	Mean ± SD				*P*-value	Controls *vs* ASD-front	Controls *vs* ASD-hyperTK	Controls *vs* ASD-sag	ASD-front vs ASD -hyperTK	ASD -front *vs* ASD-sag	ASD- hyperTK vs ASD- sag
	Controls	ASD-front	ASD-hyperTK	ASD-sag							
Short Form-36 (SF-36)											
Physical Component Summary (PCS)	50.1 ± 7.7	44.8 ± 9.6	40.5 ± 7.2	36.2 ± 8.1	**<0.001**		*	*		*	
Mental Component Summary (MCS)	55.0 ± 6.3	48.4 ± 6.7	51.7 ± 9.2	48.8 ± 10.1	**0.005**	*		*			
Visual Analog Scale (VAS)	1.3 ± 0.7	4.2 ± 2.6	6.1 ± 2.7	6.7 ± 2.6	**<0.001**	*	*	*	*	*	
Oswestry Disability Index (ODI)	3.2 ± 5.0	20.9 ± 20.2	27.2 ± 16.3	38.0 ± 16.9	**<0.001**	*	*	*		*	*
Beck’s Depression Inventory (BDI)	2.2 ± 3.8	8.7 ± 6.3	10.5 ± 7.5	11.3 ± 10.0	**<0.001**	*	*	*			

*Bold value, significant *p*-value.

### 3.4 Sitting/Standing Kinematics

Spine, pelvis, and lower limb kinematics were almost similar during both sit-to-stand (Table 2) and stand-to-sit transitions (Supplementary Table 1).

**TABLE 2:** Comparison of sit-to-stand kinematics between the four subgroups: controls, ASD-front, ASD-hyperTK, and ASD-sag.

		Mean ± SD				*P*-value	Controls vs ASD-front	Control vs ASD-hyperTK	Control *vs.* ASD-sag	ASD-front vs hyperTK	ASD -front vs ASD-sag	ASD- hyperTK vs ASD- sag
		Controls	ASD-front	ASD-hyperTK	ASD-sag							
Pelvis												
	Mean pelvic tilt (°)	15.2 ± 7.5	17.8 ± 5.6	14.1 ± 8.7	12.2 ± 9.8	**0.006**					*	
	ROM pelvic tilt (°)	37.2 ± 6.2	35.1 ± 6.0	36.1 ± 6.8	37.1 ± 7.0	0.60						
	Mean pelvic obliquity (°)	−0.1 ± 1.5	0.4 ± 1.7	0.7 ± 2.3	0.2 ± 2.5	0.26						
	ROM pelvic obliquity (°)	4.3 ± 1.9	3.7 ± 1.1	4.5 ± 2.5	5.7 ± 5.2	0.68						
	Mean pelvic rotation (°)	−0.5 ± 2.8	−0.3 ± 3.2	1.2 ± 3.3	−0.4 ± 3.8	0.10						
	ROM pelvic rotation (°)	4.9 ± 2.5	4.7 ± 1.8	5.2 ± 2.4	6.2 ± 3.1	0.20						
Hip												
	Mean hip flexion/extension (°)	57.8 ± 11.5	62.2 ± 7.7	59.2 ± 10.7	53 ± 11.2	**0.01**					*	
	ROM hip flexion/extension (°)	86.7 ± 10.6	86.6 ± 6.2	85.4 ± 14.8	81.5 ± 15.1	0.19						
Knee												
	Mean knee flexion/extension (°)	58.3 ± 10.3	61.6 ± 6.4	60.8 ± 9.9	58.8 ± 10.4	0.65						
	ROM knee flexion/extension (°)	93.9 ± 9.6	94.4 ± 7.9	89.1 ± 16.4	87.1 ± 9.3	**0.01**			*		*	
Ankle												
	Mean dorsiflexion/plantar flexion (°)	17.8 ± 8.2	17.7 ± 11.6	17.1 ± 5.6	15.7 ± 10.8	0.5						
	ROM dorsiflexion/plantar flexion (°)	22.8 ± 7.8	23.8 ± 6.7	19.9 ± 5.8	17.9 ± 5.6	**0.001**			*		*	
Head												
	Mean head flexion/extension (°)	10.5 ± 12.3	3.9 ± 14.6	2.4 ± 8.6	−3.4 ± 14.6	**0.001**		*	*			
	ROM head flexion/extension (°)	16.2 ± 7.6	19.6 ± 16.8	24.8 ± 14.6	25.6 ± 12	**0.003**			*		*	
Trunk												
	Mean trunk flexion/extension (°)	13.8 ± 6.1	12.7 ± 7.4	22.2 ± 8.0	23.9 ± 6.1	**<0.001**		*	*	*	*	
	ROM trunk flexion/extension (°)	35.4 ± 7.8	35.4 ± 9.9	41.2 ± 9.8	39.7 ± 10.6	**0.03**						
Spine segments												
	Mean flexion/extension pelvis—L3L5 (°)	19.6 ± 8.4	25.0 ± 8	24 ± 9.1	17.5 ± 10	**0.01**					*	*
	ROM flexion/extension pelvis—L3L5 (°)	41.1 ± 7.2	40 ± 7.6	41.9 ± 6.7	42 ± 8.1	0.58						
	Mean flexion/extension L1L3–L3L5 (°)	−6.8 ± 5.9	−5.7 ± 6.3	−9.1 ± 6.3	−3 ± 9.7	**0.04**						*
	ROM flexion/extension L1L3–L3L5 (°)	16.5 ± 10	14.0 ± 5.4	13.9 ± 9.2	9.1 ± 6.3	**<0.001**			*		*	*
	Mean flexion/extension T10L1–L1L3 (°)	−5.5 ± 6.4	−6.9 ± 7.1	−12 ± 9.9	−1.9 ± 11.3	**<0.001**		*				*
	ROM flexion/extension T10L1–L1L3 (°)	11.5 ± 9.3	8.3 ± 6.2	12.2 ± 5.9	9.3 ± 6.3	0.09						
	Mean flexion/extension T2T10–T10L1 (°)	17.2 ± 6.1	9.5 ± 7.4	32 ± 10.3	21.8 ± 10.9	**<0.001**	*	*		*	*	*
	ROM flexion/extension T2T10–T10L1 (°)	6.5 ± 3.1	5.9 ± 4.1	7.4 ± 4.2	9 ± 12.9	0.73						
	Mean flexion/extension C7T2–T2T10 (°)	17.7 ± 6.5	21.6 ± 8.6	30.4 ± 9.6	25.2 ± 8	**<0.001**		*	*	*		
	ROM flexion/extension C7T2–T2T10 (°)	11 ± 6.5	12.5 ± 5.7	18.1 ± 11.9	14.2 ± 11.2	**0.04**		*				

ROM, range of motion.

*Bold value, significant *p*-value.

During sitting and standing movements, when compared with controls, ASD-sag presented with a decreased mean pelvic anteversion (dynamic pelvic tilt: 12.2 vs 15.2°, *p* = 0.006), mean hip flexion (53.0 vs 62.2°, *p* = 0.006), and knee and ankle sagittal ROM (87.1 vs 93.9°, *p* = 0.01 and 17.9 vs 22.8°, *p* < 0.001 resp.). At the spinal segmental level, ASD-sag presented with a decreased lumbar sagittal ROM when compared with the other subgroups (L1L3–L3L5: 9.1 vs 16.5° in controls, *p* < 0.001).

ASD-hyperTK showed similar lower limb kinematics compared with controls. However, at the spinal level, they had an increased dynamic lumbar lordosis (mean L1L3–L3L5: −9.1 vs −6.8° in controls, *p* = 0.04), increased extension at the thoracolumbar junction (mean T10L1–L1L3: −12.4 vs −5.5°, *p* < 0.001), and more flexed thoracic segments (mean T2T10–T10L1: 32.0 vs 17.2°, mean C7T2–T2T10: 30.4 vs 17.7°, both *p* < 0.001) when compared with controls. They also showed increased sagittal ROM both at the thoracolumbar junction (ROM T10L1–L1L3: 16.3 *vs.* 9.8°, *p* = 0.004) and the upper thoracic level (ROMC7T2–T2T10: 18.1 vs 11.0°, *p* = 0.04).

Both ASD-sag and ASD-hyperTK maintained a flexed trunk during sit-to-stand and stand-to-sit movements (mean trunk flexion/extension: 28.6 and 25.1° resp., vs 15.9° in controls, *p* < 0.001) along with an extended head (mean head flexion/extension: −13.8 and −5.3° resp., vs 8.9° in controls, *p* < 0.001). Both groups also had increased trunk and head sagittal ROM (44.2 and 43.5° vs 34.6°; 40.9 and 36.9° vs 16.9° resp., both *p* < 0.001).

ASD-front had sitting and standing kinematics that were comparable with controls.

**FIGURE 4:**
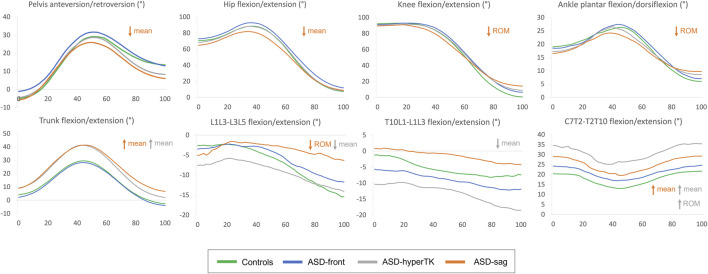
Average kinematic waveforms for each subgroup during the sit-to-stand movement cycle (normalized between 0 and 100%). ROM, range of motion. Statistically significant differences during sit-to-stand only have been represented.

Waveforms of major kinematic differences between ASD subgroups and controls are displayed in Figure 4.

### 3.5 Univariate Analysis

Altered sitting and standing kinematics were significantly correlated with standing radiographic parameters and HRQOL scores. In particular, lumbar ROM and hip flexion were negatively correlated to SVA (*r* = −0.26 and *r* = −0.25 resp., *p* = 0.004 and *p* = 0.005 resp.), PT (*r* = −0.29 and *r* = −0.37 resp., *p* = 0.001 and *p* < 0.001 resp.), and PI-LL mismatch (*r* = −0.31 and *r* = −0.32 resp., both *p* < 0.001). Mean trunk flexion as well as trunk and head sagittal ROM were positively correlated to TK (*r* = 0.45, *r* = 0.28, and *r* = 0.28 resp., all *p* = 0.001) and PT (*r* = 0.38, *r* = 0.21, and *r* = 0.21 resp., *p* < 0.001, *p* = 0.002, and *p* = 0.002 resp.).

**FIGURE 5:**
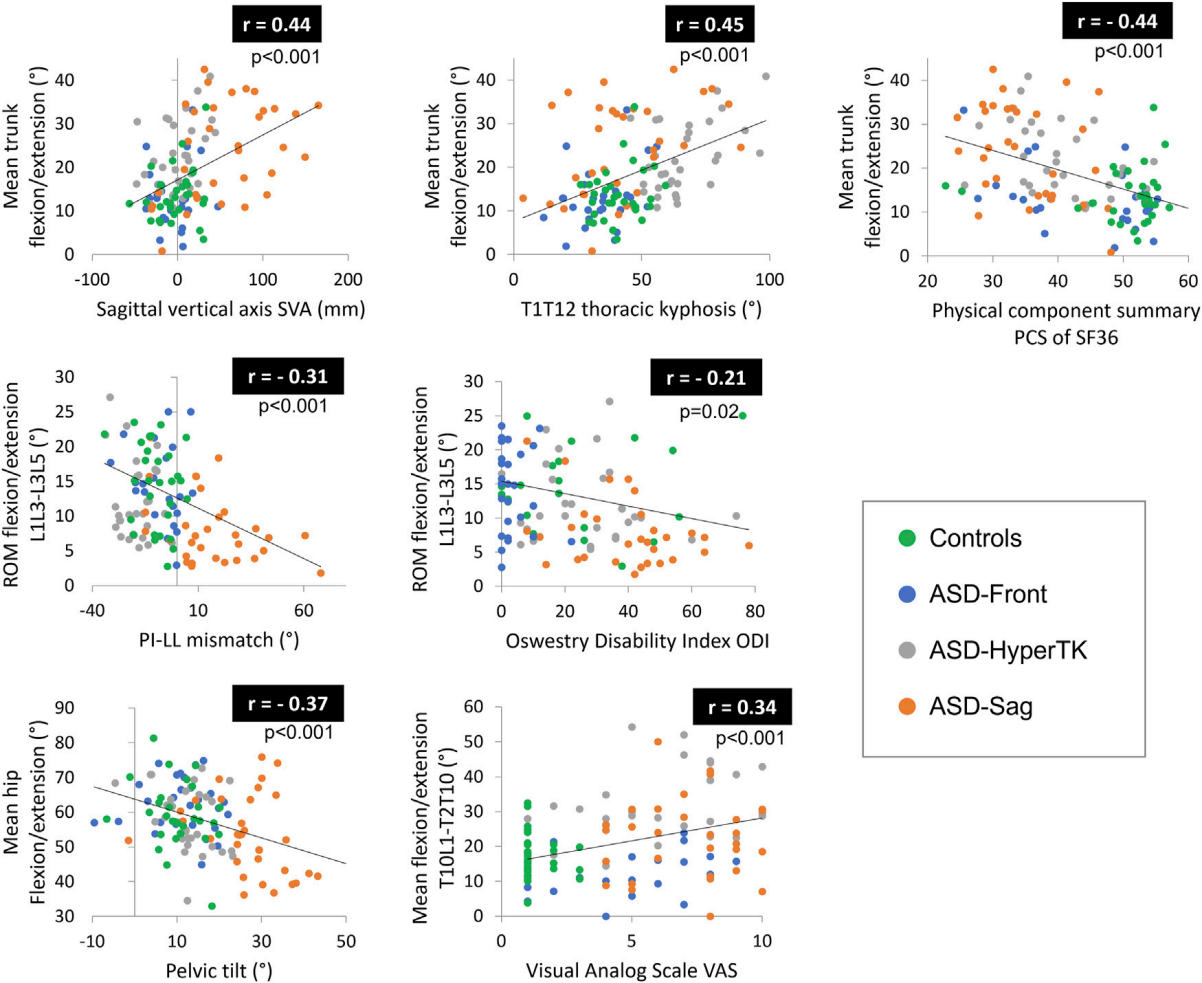
Correlations between altered kinematic parameters and both radiographic parameters and health-related quality of life (HRQOL) scores. ROM, range of motion.

Furthermore, mean thorax flexion as well as trunk and head sagittal ROM were negatively correlated to PCS (*r* = −0.44, *r* = −0.27, and *r* = −0.26 resp., *p* < 0.001, *p* = 0.002, and *p* = 0.004 resp.) but positively correlated to VAS (*r* = 0.44, *r* = 0.27, and *r* = 0.30 resp., *p* < 0.001, *p* = 0.003, and *p* = 0.001 resp.), ODI (*r* = 0.46, *r* = 0.30, and *r* = 0.34 resp., all *p* < 0.001), and BDI (*r* = 0.31, *r* = 0.33, and 0.32 resp., all *p* < 0.001) (Figure 5).

### 3.6 Determinants of Kinematic Alterations

The multivariate analysis showed that even after controlling for demographic factors, kinematic alterations could be determined by spino-pelvic parameters:

Dynamic trunk flexion was determined by TK and PI-LL mismatch (adj. *R* ^2^ = 0.44; *β* = 0.55 and *β* = 0.35 resp., *p* < 0.001). Dynamic L1L3–L3L5 lumbar lordosis was determined by PI-LL mismatch and knee flexion (adj. *R* ^2^ = 0.29; β = 0.47 and β = 0.19 resp., *p* < 0.001). Dynamic T2T10–T10L1 thoracic kyphosis was determined by TK and PT (adj. *R* ^2^ = 0.62; *β* = 0.44 and *β* = 0.15 resp., *p* < 0.001).

Lumbar sagittal ROM was determined by PI-LL mismatch (adj. *R* ^2^ = 0.13; *β* = −0.23, *p* < 0.001). Head sagittal ROM was determined by TK and PT (adj. *R* ^2^ = 0.17; *β* = 0.28 and *β* = 0.21 resp., *p* < 0.001).

## 4 Discussion

Patients with adult spinal deformity (ASD) are known to have quality of life (HRQOL) alterations and functional limitations (Pellisé et al., 2015; Christopher Kieser and Wyatt, 2019). While gait adaptations in ASD have been previously described in the literature (Kawkabani et al., 2021), alterations in other daily life activities, such as sitting and standing, have been poorly characterized. Furthermore, ASD represent a complex and heterogeneous entity differing in radiographic alterations, HRQOL scores, and even outcome after surgical intervention (Bess et al., 2016; Bakhsheshian et al., 2017; Yang et al., 2019). It is therefore crucial to subdivide the ASD population depending on the type of spinal deformity to better understand their motion alterations and provide appropriate treatment. This study recruited 93 ASD subjects with different types of spinal deformity and 31 controls to describe kinematic alterations in each ASD subgroup, divided according to their spinal deformity, during sit-to-stand and stand-to-sit movements, and further investigate the relationships between these kinematic changes and radiographic parameters as well as HRQOL scores.

The ASD population included in this study was found to have standing radiographic alterations comparable with those described in previous studies (Le Huec et al., 2019). ASD-sag had a loss of lumbar lordosis with a forward shift of the trunk. This resulted in increased pelvic retroversion and knee flexion to maintain their center of gravity above their feet. ASD-hyperTK presented with a thoracic hyperkyphosis that was compensated by an increase in lumbar lordosis and cervical lordosis without the need for other compensating mechanisms in the pelvis or lower limbs. ASD-front presented only with a coronal Cobb angle that did not affect their sagittal balance. Furthermore, ASD patients had significant pain (higher VAS) and HRQOL score alterations both on the physical (lower PCS and greater ODI) and mental components (lower MCS and higher BDI), as reported in previous studies (Pellisé et al., 2015; Diebo et al., 2018b, 2018a). These alterations were more pronounced in ASD-sag and to a lesser degree in ASD-hyperTK and ASD-front.

During sit-to-stand, the control group showed kinematics similar to those described in previous studies (Schenkman et al., 1990; Roebroeck et al., 1994; Tully et al., 2005). Initial forward propulsion of the trunk was mainly achieved by hip flexion. This acquired flexion momentum allowed them to achieve lift-off, while starting to extend their knees. At the same time, controls started to gradually increase ankle dorsiflexion, further projecting their trunk anteriorly. Afterwards, subjects simultaneously extended their lumbar spines, hips, knees, and ankles to reach the erect standing position. The thoracic spine underwent an initial compensatory extension followed by flexion during the transition to the erect phase. Horizontal gaze was maintained by an initial extension of the head and neck followed by flexion. Stand-to-sit kinematics showed mostly the same sequence of events, in reverse.

During sit-to-stand and stand-to-sit movements, ASD-sag showed altered pelvis and lower limb parameters. They had a more retroverted pelvis during motion, a previously described compensation mechanism in the standing position that allowed them to maintain their center of gravity over their base of support (Le Huec et al., 2019). They also presented with a limited hip flexion capacity and decreased mobility in their knees and ankles.

Since hip flexion is measured as the motion of the femur relative to the pelvis, pelvic retroversion in ASD-sag, which is the case here, could result in an apparent decrease of hip flexion. However, if the decreased mean hip flexion was solely due to pelvic retroversion, one would expect hip flexion to be also decreased during the final stage of motion when the subject assumes the standing position where pelvic retroversion is prominent as shown in Figure 4 in this group. On further examination of the corresponding kinematic curve (Figure 4), peak hip flexion seems to be the most affected in ASD-sag. This corroborates results by Bailey et al. who had previously demonstrated that ASD patients had a decreased peak of hip flexion during sit-to-stand, along with an increased energy expenditure at this level (Bailey et al., 2019). Limitation in hip flexion could serve to prevent additional forward bending of the trunk in the sitting position and during the transition from sitting to standing, since ASD-sag already present with an increased trunk flexion during motion.

The decreased lower limb mobility during sitting and standing has also been described during walking, especially at the level of the knees. As mentioned earlier, in the static standing position, knee flexion acts as a compensation mechanism that repositions the center of gravity above the feet. The same mechanism is also maintained during walking, therefore limiting knee extension and leading to decreased knee ROM during gait and eventually decreased step length (Kawkabani et al., 2021; Severijns et al., 2021). However, in the static sitting position, the height of the seat was adjusted so that all individuals had the same initial knee flexion of 90°. Therefore, the decrease in knee ROM seen during sit-to-stand in ASD-sag only reflects the lack of knee extension in the standing position, as shown in the final stage of the knee kinematic curve (Figure 4).

At the level of the ankles, ASD-sag presented with limited peak dorsiflexion, which could act to prevent excessive forward bending of the trunk when transitioning from the sitting to the standing position, similar to the limitation in peak hip flexion. Furthermore, they had increased dorsiflexion in the final stage of the motion, while assuming the static standing position. Increased ankle dorsiflexion has previously been described as a compensation mechanism associated with knee flexion in ASD (Ferrero et al., 2016). Both decreased peak dorsiflexion and increased dorsiflexion in the final standing stage seem to explain the decreased ankle ROM seen in ASD-sag.

At the segmental level of the spine, ASD-sag also showed a decrease in lumbar (L1L3–L3L5) mobility during sit-to-stand and stand-to-sit. This is in agreement with other studies that showed a lesser variation of radiographic lumbar lordosis between the standing and sitting positions in ASD (Buckland et al., 2020). ASD-sag showed a fixed and decreased lumbar lordosis during the whole sit-to-stand in contrast to the other groups who were able to restore normal lumbar lordosis during standing (Figure 4). Indeed, loss of lumbar lordosis is regarded as the *primum movens* of the degenerative sagittal deformity seen in ASD (Le Huec et al., 2019).

ASD-hyperTK did not present significant differences in pelvic or lower limb kinematics compared with controls. This further highlights the fact that in ASD-hyperTK, no alteration or compensatory mechanism is seen at the level of the pelvis and lower limbs since thoracic hyperkyphosis is compensated by lumbar and cervical hyperlordosis when analyzing standing radiographic posture. However, these more pronounced spinal curvatures were reflected in spine segmental kinematics as increased dynamic lumbar (L1L3–L3L5) lordosis, increased extension at the thoracolumbar junction (T10L1–L1L3) as well as a more flexed thoracic segments (C7T2–T2T10 and T2T10–T10L1) when compared with controls. These increased curvatures required an increased mobility of the spine during motion. This was apparent at the thoracolumbar junction (T10L1–L1L3) as well as the upper thoracic spine (C7T2–T2T10). This finding confirms other observations showing that in the thoracic spine, the highest segmental ROM in the sagittal plane occurred at the upper-thoracic and thoracolumbar levels (Morita et al., 2014).

Overall, both ASD-sag and ASD-hyperTK maintained a flexed trunk during sit-to-stand and stand-to-sit. This forward inclination of the trunk is a characteristic feature of ASD in the standing position and has been shown to persist during gait in ASD individuals (Kawkabani et al., 2021). Furthermore, this was also described in other studies where peak dynamic SVA during sit-to-stand was shown to be increased in ASD (Bailey et al., 2019). This forward increase could also serve to increase stability by facilitating the transition of their center of gravity from the seat’s wide base to the narrower base of their feet (Hughes et al., 1994). In fact, sit-to-stand transition requires acquiring initial momentum through combined hip and lumbar flexion, which is then transferred to the trunk allowing the propulsion of the subject from the seat. This requires high levels of neuromuscular control, which might be altered in individuals with spinal deformity and compensating mechanisms (Arima et al., 2018; Laratta et al., 2019). An alternative strategy described as a “stabilization” strategy involves moving the center of gravity above the base of the feet first and then extending the lower limbs and trunk to assume the standing position (Hughes et al., 1994). The forward increase of the trunk can move the center of gravity easily to the narrow base of the feet without needing to go through an unstable phase of momentum transfer. As shown previously, different strategies were adopted as compensatory mechanisms for the forward bending of the trunk. ASD-sag who presented with a more rigid spine recruited compensatory mechanisms in their lower limbs with a reduced peak hip flexion and reduced ankle dorsiflexion. ASD-hyperTK compensated by increasing the mobility of their upper thoracic and thoracolumbar spines. Both strategies prevented excessive trunk flexion and loss of balance.

To maintain a horizontal gaze, ASD-sag and ASD-hyperTK needed to further extend their head during motion, as described in previous studies where radiographic cervical lordosis was shown to increase parallel to the increase in SVA (Diebo et al., 2016). The findings of this study showed that these modifications were associated with a compensatory increase in mobility of the trunk and head in both ASD-sag and ASD-hyperTK.

ASD-front had similar kinematics compared with controls. This reflects the fact than an isolated scoliosis does not affect sagittal balance and therefore motion during sit-to-stand and stand-to-sit, which mostly occur in the sagittal plane (Gilleard et al., 1999).

The kinematic modifications observed in the ASD subgroups were correlated to the radiographic alterations. In particular, an increase in radiographic PT, SVA, and PI-LL, the main altered parameters in ASD-sag, was correlated to a decreased lumbar mobility and decreased hip flexion. Mean trunk flexion as well as trunk and head sagittal ROM were positively correlated to TK, the main driver of the deformity in ASD-hyperTK, and PT, the main compensating mechanism in ASD-sag. Furthermore, kinematic alterations were also correlated to the HRQOL score alterations in ASD.

Our study had several limitations. First, even though sex distribution did not significantly differ between ASD subjects and controls, sex ratios were not identical (ASD: 71F/22M vs controls: 18F/13M). Furthermore, both groups covered a wide range of age groups. Demographics such as age and sex are known to affect mobility in asymptomatic subjects (Zhou et al., 2020a; 2020b). However, even after controlling for demographics as confounding variables, kinematics were still different between groups. Second, some subjects with severe deformities were not able to stand up without assistance. They were therefore allowed to lean on their thighs during sit-to-stand transition. While this strategy might affect sit-to-stand kinematics, excluding these subjects would have resulted in a selection bias where only milder deformities are included, potentially masking the differences between subgroups. Furthermore, the “hands on knees” strategy was allowed since ground reaction forces and average events times were shown to be similar to rising from the seat with arms free or with crossed arms (Etnyre and Thomas, 2007). Finally, errors inherent to the marker model and tracking system could affect the validity of our data, especially in the spinal segment. For instance, fixed segments were used to describe spine kinematics, instead of a subject-specific model. However, this model has already been studied in the literature and was shown to have good intra-subject repeatability (Leardini et al., 2011). Furthermore, the values of kinematic parameters for the control group in this study were similar to those initially described by Leardini et al.

Nevertheless, this study described patterns of movement alterations that are specific to each subgroup of ASD deformity and that might have clinical implications. ASD with a sagittal malalignment had decreased lumbar mobility during sit-to-stand. Surgical fixation of the spine might lead to more altered spine kinematics, requiring further compensating mechanisms in the adjacent spinal segments, pelvis, and lower limbs. In ASD with an isolated thoracic hyperkyphosis, increased mobility of the thoracic spine at both its upper and lower ends could serve as a compensation mechanism during sit-to-stand. Surgical correction of the deformity could also increase the rigidity of these segments, therefore limiting the compensatory ability of the spine and recruiting additional compensation mechanisms in the pelvis and lower limbs. It is still not clear if surgery is able to restore normal kinematics in ASD. Bailey et al. have shown that peak trunk flexion was reduced and peak hip flexion was increased postoperatively, along with a decrease in lumbar and hip torques. However, energy expenditure at the level of the knees was increased and spine kinematics were not studied (Bailey et al., 2019). In other studies, alterations in limb kinematics and spatio-temporal parameters during gait were not corrected postoperatively (Severijns et al., 2021). This highlights the need for future studies that would be able to assess the effect of surgery on daily life activities and that could determine which subset of ASD patients might benefit the most from surgical interventions.

In conclusion, both ASD with sagittal malalignment and those with an isolated hyperkyphosis had a flexed trunk attitude, compensated by an extended head and an increased mobility of their trunk and heads during the sit-to-stand and stand-to-sit movements. ASD-sag had limited lumbar and lower limb mobility similar to the alterations seen during walking. ASD-hyperTK had a flexed attitude at the thoracic spinal segments compensated by an increased extension in the lumbar and thoracolumbar segments, along with an increased mobility at the upper-thoracic and thoracolumbar junction. These kinematic alterations were correlated to radiographic spino-pelvic malalignment and HRQOL deteriorations. Future studies should address whether spinal corrective surgery or physical reeducation are able to improve sitting and standing kinematics in ASD patients and therefore their quality of life.

## Data Availability

The raw data supporting the conclusions of this article will be made available by the authors, without undue reservation.
