# Supplementary material for: Alteration of the Sitting and Standing Movement in Adult Spinal Deformity
Source: Front Bioeng Biotechnol. 2022 Jan 13;9:751193. doi: 10.3389/fbioe.2021.751193 (PMC8792509; doi:10.3389/fbioe.2021.751193)
Supplement: Supplementary file 1 [file DataSheet1.DOCX]

Table 1: Comparison of stand-to-sit kinematics between the 4 subgroups: controls, ASD-front, ASD-hyperTK and ASD-sag

|  |  | Mean ± SD | | | | | | | | | | | | p-value | Controls  vs ASD-front | Controls vs ASD-hyperTK | Controls  vs ASD-sag | ASD-front  vs hyperTK | ASD-front  vs ASD-sag | ASD-hyperTK vs ASD-sag |
| --- | --- | --- | --- | --- | --- | --- | --- | --- | --- | --- | --- | --- | --- | --- | --- | --- | --- | --- | --- | --- |
|  |  | Controls | | | ASD-front | | | ASD-hyperTK | | | ASD-sag | | |  |  |  |  |  |  |  |
| Pelvis | |  |  |  |  |  |  |  |  |  |  |  |  |  |  |  |  |  |  |  |
|  | Mean pelvic tilt (°) | 16.5 | ± | 7.4 | 20.0 | ± | 7.3 | 15.9 | ± | 9.1 | 15.8 | ± | 11.2 | 0.07 |  |  |  |  |  |  |
|  | ROM pelvic tilt (°) | 39.6 | ± | 6.8 | 38.0 | ± | 7.2 | 39.7 | ± | 6.7 | 39.5 | ± | 9.1 | 0.88 |  |  |  |  |  |  |
|  | Mean pelvic obliquity (°) | 0.0 | ± | 1.4 | 0.2 | ± | 1.4 | 0.6 | ± | 2.0 | 0.4 | ± | 2.8 | 0.70 |  |  |  |  |  |  |
|  | ROM pelvic obliquity (°) | 4.7 | ± | 2.2 | 5.0 | ± | 3.1 | 4.7 | ± | 2.1 | 6.6 | ± | 6.2 | 0.59 |  |  |  |  |  |  |
|  | Mean pelvic rotation (°) | -0.8 | ± | 2.8 | -1.0 | ± | 2.6 | 0.8 | ± | 2.4 | 0.6 | ± | 4.9 | **0.05** |  |  |  |  |  |  |
|  | ROM pelvic rotation (°) | 4.9 | ± | 2.1 | 6.0 | ± | 2.2 | 6.3 | ± | 3.1 | 6.3 | ± | 2.5 | 0.12 |  |  |  |  |  |  |
| Hip | |  |  |  |  |  |  |  |  |  |  |  |  |  |  |  |  |  |  |  |
|  | Mean hip flexion/extension (°) | 63.9 | ± | 8.7 | 65.8 | ± | 8.0 | 61.4 | ± | 9.6 | 59.1 | ± | 11.9 | **0.04** |  |  |  |  |  |  |
|  | ROM hip flexion/extension (°) | 84.0 | ± | 8.9 | 86.1 | ± | 8.6 | 84.9 | ± | 15.3 | 80.8 | ± | 14.1 | 0.26 |  |  |  |  |  |  |
| Knee | |  |  |  |  |  |  |  |  |  |  |  |  |  |  |  |  |  |  |  |
|  | Mean knee flexion/extension (°) | 63.9 | ± | 9.8 | 61.4 | ± | 8.9 | 62.8 | ± | 9.9 | 61.5 | ± | 7.7 | 0.56 |  |  |  |  |  |  |
|  | ROM knee flexion/extension (°) | 91.8 | ± | 8.6 | 92.6 | ± | 8.7 | 91.3 | ± | 8.7 | 86.2 | ± | 8.6 | **0.02** |  |  |  |  | * |  |
| Ankle | |  |  |  |  |  |  |  |  |  |  |  |  |  |  |  |  |  |  |  |
|  | Mean dorsiflexion/plantar flexion (°) | 19.1 | ± | 8.7 | 18.4 | ± | 5.0 | 17.9 | ± | 6.1 | 17.6 | ± | 5.5 | 0.94 |  |  |  |  |  |  |
|  | ROM dorsiflexion/plantar flexion (°) | 21.1 | ± | 7.1 | 22.2 | ± | 5.2 | 18.5 | ± | 6.0 | 19.8 | ± | 13.5 | **0.02** |  |  |  |  | * |  |
| Head | |  |  |  |  |  |  |  |  |  |  |  |  |  |  |  |  |  |  |  |
|  | Mean head flexion/extension (°) | 8.9 | ± | 12.8 | -2.9 | ± | 19.2 | -5.3 | ± | 14.5 | -14 | ± | 19.4 | **<0.001** | * | * | * |  |  |  |
|  | ROM head flexion/extension (°) | 16.9 | ± | 8.4 | 28.7 | ± | 24.6 | 36.9 | ± | 23.3 | 40.9 | ± | 21.7 | **<0.001** |  | * | * |  | * |  |
| Trunk | |  |  |  |  |  |  |  |  |  |  |  |  |  |  |  |  |  |  |  |
|  | Mean trunk flexion/extension (°) | 15.9 | ± | 5.7 | 15.5 | ± | 9.4 | 25.1 | ± | 10.7 | 28.6 | ± | 12.6 | **<0.001** |  | * | * | * | * |  |
|  | ROM trunk flexion/extension (°) | 34.6 | ± | 7.2 | 39.2 | ± | 13.5 | 43.5 | ± | 10.4 | 44.2 | ± | 11.7 | **0.001** |  | * | * |  |  |  |
| Spine segments | |  |  |  |  |  |  |  |  |  |  |  |  |  |  |  |  |  |  |  |
|  | Mean flexion/extension pelvis-L3L5 (°) | 22.8 | ± | 8.2 | 27.9 | ± | 9.7 | 26.5 | ± | 10.5 | 22.8 | ± | 11.7 | 0.08 |  |  |  |  |  |  |
|  | ROM flexion/extension pelvis-L3L5 (°) | 44.1 | ± | 6.7 | 42.7 | ± | 8.8 | 47.5 | ± | 11.9 | 45.1 | ± | 10.5 | 0.47 |  |  |  |  |  |  |
|  | Mean flexion/extension L1L3-L3L5 (°) | -7.6 | ± | 6.0 | -6.3 | ± | 6.1 | -9.1 | ± | 6.4 | -3.6 | ± | 9.9 | 0.10 |  |  |  |  |  |  |
|  | ROM flexion/extension L1L3-L3L5 (°) | 13.1 | ± | 6.3 | 13.4 | ± | 5.5 | 14.6 | ± | 9.4 | 9.3 | ± | 8.1 | **0.001** |  |  | * |  | * | ***** |
|  | Mean flexion/extension T10L1-L1L3 (°) | -6.1 | ± | 6.2 | -7.6 | ± | 6.8 | -12 | ± | 9.8 | -2.9 | ± | 11.6 | **0.001** |  | * |  |  |  | ***** |
|  | ROM flexion/extension T10L1-L1L3 (°) | 9.8 | ± | 5.0 | 9.4 | ± | 5.7 | 16.3 | ± | 12.8 | 8.6 | ± | 6.0 | **0.004** |  |  |  |  |  | ***** |
|  | Mean flexion/extension T2T10-T10L1 (°) | 17.6 | ± | 6.5 | 13.8 | ± | 7.2 | 31.1 | ± | 10.2 | 21.1 | ± | 11.1 | **<0.001** |  | * |  | * | * | ***** |
|  | ROM flexion/extension T2T10-T10L1 (°) | 5.7 | ± | 2.5 | 8.2 | ± | 4.9 | 8.1 | ± | 6.6 | 12.4 | ± | 18.7 | 0.16 |  |  |  |  |  |  |
|  | Mean flexion/extension C7T2-T2T10 (°) | 17.9 | ± | 6.1 | 21.6 | ± | 8.9 | 30.4 | ± | 9.6 | 25.2 | ± | 8.3 | **<0.001** |  | * | * | * |  |  |
|  | ROM flexion/extension C7T2-T2T10 (°) | 9.8 | ± | 4.1 | 12.2 | ± | 7.1 | 14.2 | ± | 9.5 | 11.6 | ± | 8.8 | 0.23 |  |  |  |  |  |  |

ROM: range of motion

*, bold value: significant p-value
